# Applying Box–Behnken Design for Formulation and Optimization of PLGA-Coffee Nanoparticles and Detecting Enhanced Antioxidant and Anticancer Activities

**DOI:** 10.3390/polym14010144

**Published:** 2021-12-31

**Authors:** Nouran S. Sharaf, Amro Shetta, Jailan E. Elhalawani, Wael Mamdouh

**Affiliations:** Department of Chemistry, School of Sciences and Engineering, The American University in Cairo (AUC), AUC Avenue, P.O. Box 74, New Cairo 11835, Egypt; nouransharaf@aucegypt.edu (N.S.S.); amrpharma@aucegypt.edu (A.S.); jailanessam@aucegypt.edu (J.E.E.)

**Keywords:** coffee extract, nanoparticles, PLGA, antioxidant, cytotoxicity

## Abstract

In an attempt to prove biological activity enhancement upon particle size reduction to the nanoscale, coffee (Cf) was chosen to be formulated into poly(lactic-*co*-glycolic) acid (PLGA) nanoparticles (NPs) using the single emulsion-solvent evaporation (SE-SE) method via Box–Behnken Design (BBD) to study the impact of certain process and formulation parameters on the particle size and size homogeneity, surface stability and encapsulation efficiency (EE%). The coffee-loaded PLGA (PLGA-Cf) NPs were characterized by different methods to aid in selecting the optimum formulation conditions. The desirable physicochemical characteristics involved small particle sizes with an average of 318.60 ± 5.65 nm, uniformly distributed within a narrow range (PDI of 0.074 ± 0.015), with considerable stability (Zeta Potential of −20.50 ± 0.52 mV) and the highest EE% (85.92 ± 4.01%). The antioxidant and anticancer activities of plain PLGA NPs, pure Cf and the optimum PLGA-Cf NPs, were evaluated using 2,2-Diphenyl-1-picryl-hydrazyl (DPPH) and 3-(4,5-dimethylthiazol-2-yl)-2,5-diphenyltetrazolium bromide (MTT) assays, respectively. As a result of nano-encapsulation, antioxidant activity was enhanced by 26.5%. Encapsulated Cf showed higher anticancer potency than pure Cf against different cancerous cell lines with an increase of 86.78%, 78.17%, 85.84% and 84.84% against MCF-7, A-549, HeLa and HepG-2, respectively. The in vitro release followed the Weibull release model with slow and biphasic release profile in both tested pH media, 7.4 and 5.5.

## 1. Introduction

Coffee (Cf, family *Rubiaceae*), as a drink, occupies an exceptionally respectable position that no other beverage has reached. This is owing to its attractive flavor and aroma, cognition stimulation in addition to its relative safety for human consumption (to a certain limit) [1]. Therefore, it participates in different sectors of food, pharmaceutical and cosmetic industries. Moreover, Cf was anciently used in several cultures in their traditional medicine [2], and scientifically proved thereafter to possess a wide variety of health effects (ranging from antioxidant, antibacterial, antifungal, anticancer, anti-inflammatory, neuroprotective, etc.) which arise from a huge collection of naturally occurring bioactive compounds of which polyphenols, phenolic acids (e.g., chlorogenic acids (CGAs), caffeic, coumaric and ferulic acids, etc.) and alkaloids (caffeine and trigonelline) have an evident existence and particular attention. These biologically active constituents vary in their ratios with respect to the bean roasting degrees (green, light, medium and dark roasted brews) [3,4], the plant species (*Coffee arabica* and *Coffee canephora* (known as Robusta)) [3,4] and the plant origins (Brazil, Vietnam, Colombia, Indonesia, etc.) [5,6,7,8]. In addition, it is worth mentioning that besides the natural bioactive components, there are other active substances that develop upon roasting the beans by binding to the existing phenolic compounds, called Maillard Reaction Products (MRPs, e.g., melanoidins), and were evidenced to possess certain biological effects [9,10,11].

In light of our study, coffee was reported, as a whole plant extract and as isolated ingredients, to have well-established antioxidant [8,9,10] and anticancer activities [11,12] which encouraged its use as a natural alternative to synthetic drugs or as an additive to improve the potentiality of other drugs in terms of delivery enhancement, dose reduction and side effect minimization. Usually, a remarkable antioxidant activity potentially confers further biological activity enhancement such as the anticancer and anti-rheumatic effects since an antioxidant neutralizes the free radicals and oxidative products (reactive oxygen species (ROS) and reactive nitrogen species (RNS) generated and accumulated in the human cells and eventually leads to cellular function impairment and the evolution of degenerative diseases such as cancer [13,14].

Nevertheless, the health benefits of the polyphenolics were found largely restricted due to multiple intrinsic properties such as the unpleasant oral taste, low water solubility, poor intestinal absorption (low bioavailability), and the overall in vivo and in vitro instability. Therefore, nanotechnology is one of the recently evidenced state-of-the-art solutions that provided superior pharmaceutical rewards to overcome these limitations [15].

Particularly, nano-encapsulation into polymeric NPs is a highly rich field of experimental research since the polymeric drug delivery nano-systems possess high structural integrity and high stability during storage and when ingested in addition to their easy design, preparation and functionalization and their capability of enhancing water solubility and controlling the release of the contained material [16]. In addition, incorporation of plant-based active compounds into polymeric NPs and their exploitation as adjuvant and/or synergistic therapy to existing drugs has reported promising achievements in providing treatments with low drug dosage to ensure safety and sustain immunity [17].

Polymeric NPs made from biodegradable and biocompatible polymers (biopolymers) have been applied to encapsulate a huge variety of molecules. PLGA is one of the highly evidenced biopolymers in carrying and protecting a drug, targeting its delivery, and controlling its release. It is readily hydrolyzed in the body into its original building blocks (lactic and glycolic acids) which are already endogenous monomers and normally metabolized in the human body with insignificant systemic toxicity. Accordingly, it has been approved by the FDA as a biomaterial for human use in a wide range of applications in the pharmaceutical, diagnostic and therapeutic field [18]. As reviewed in previous literature, the most implemented method for fabricating the PLGA-based NPs involved the single emulsion-solvent evaporation (SE-SE) which is best suited for encapsulating the hydrophobic drugs in the form of oil/water (o/w) emulsions. This procedure is derived from the generalized and basic technique: the emulsification-solvent evaporation (ESE) [19]. Furthermore, polyvinyl alcohol (PVA) is a very common emulsifier/stabilizer that is extensively used in formulating PLGA-based nano-systems, due to its capability of formulating stable and homogeneously distributed small-sized NPs [20]. There are several attractive features that made PVA an ideal candidate, such as its high water-solubility, biocompatibility, biodegradability, relative safety and high bioavailability upon oral [21], topical [22] and intravenous administration [23,24].

Considering our study, it is important to mention that there has not yet been any attempt to encapsulate extracts of Cf beans in a PLGA-based nano-system. However, it was first encapsulated in 2016 [25] as a Cf residue extract into oxidized tapioca starch through coacervation but the obtained size was beyond the nano-range, reaching up to 1699.3 µm. Another group of researchers published a study in July 2019 [26] that involved formulating Cf extract into solid lipid nanoparticles (SLNs) via melt emulsification-high pressure homogenization method which were intended to be taken up by the lymphatic route. Another research group published a study that comprised encapsulating green Cf beans extract within maltodextrin through spray drying technique, applying different polymer-to-drug ratios (*g*/*g*) at different temperatures to investigate their effects on four different responses: the moisture percentage of NPs, product yield, EE%, TPC and antioxidant activity (by ABTS assay) [27]. The most recent experimental study was held early in 2020 [28] to investigate the potentiality of Arabica Cf grounds to be used as an active pharmaceutical ingredient, achieved via ball milling combined with ultrasonication. The produced NPs were poly-dispersed with a size of 396.0 nm and the particles were found as irregular granules under the SEM [28]. Moreover, the volatile oil from pressed roasted beans was experimented on by a group of researchers who attempted to stabilize its flavoring compounds by encapsulation through the ESE method [29].

Accordingly, we attempted here to evidence the hypothesis of extract-encapsulated NPs that could improve the physicochemical properties and enhance the bioactivity of the Cf extract in terms of the antioxidant and anticancer activities. Therefore, we designed biopolymeric nanoparticulate Cf carriers made of PLGA with controlled diameter, size distribution, surface charge, EE and loading capacity (LC).

## 2. Materials and Methods

### 2.1. Materials

PLGA (Mw 38,000–54,000), Polyvinyl alcohol (PVA) (30,000–70,000 Mw), and 2,2-Diphenyl-1-picryl-hydrazyl (DPPH) were purchased from Sigma Aldrich, Hamburg, Germany. Dichloromethane (DCM) and Folin–Ciocalteau (F-C) were purchased from Fisher Chemical, Hampton, Ireland. The medium-roasted powdered Colombian Cf was purchased from international brand, Starbucks, (Cairo), Egypt. Gallic acid was purchased from (Merck KGaA, Darmstadt, Germany). For the cytotoxicity assay, all the cell lines were obtained from VACSERA Tissue Culture Unit, Cairo, Egypt.

### 2.2. Methods

First of all, this study comprised three main experimental stages, starting with the extraction of Cf, followed by the preparation of PLGA-Cf NPs (with the aid of BBD), characterization and optimization of the preparation conditions where the optimally obtained NPs of this stage were subjected to a final optimization stage where the initial extract amount was varied. Lastly, the in vitro evaluation (of antioxidant and cytotoxic activities) was carried out on the PLGA-Cf NPs obtained from the third stage, having the most desirable characteristics.

Three-factor, three-level BBD was implemented for optimization of PLGA-Cf NPs. The BBD includes 15 experimental runs with three replicated center points. The independent factors were PVA concentration (X_1_), homogenization speed (X_2_), and homogenization time (X_3_). The three variables were varied at three levels: low (coded as −1), middle (coded as 0) and high (coded as 1). The ratio of Cf extract to PLGA was kept constant (0.1:1 *w*/*w*). The responses or dependent variables studied were particle size (Y_1_), Zeta Potential (ZP) (Y_2_), poly disperse index (PDI) (Y_3_), encapsulation efficiency (EE%) (Y_4_), and loading capacity percentage (LC%) (Y_5_). Selected ranges are shown in Table 1.

Generally, we employed the BBD since it is commonly used to construct higher-order response surfaces applying minimal number of runs compared to a normal factorial technique. Altogether with the central composite technique, they suppress certain runs in order to maintain the higher-order surface prospects.

### 2.3. Optimization of PLGA-Cf NPs by Multiple Response

In this study, all three responses were simultaneously optimized by using the general linear scale desirability function. The optimum formulation was selected based on the criteria of attaining the minimum particle size and PDI, whereas maximum ZP, EE%, and LC% as shown in (Table 1). New formulations were prepared according to the optimized independent variables. The Cf extract-to-PLGA polymer ratio was changed to study its impact on the responses that were previously studied in phase one. Accordingly, the resulted samples were denoted as PLGA-Cf-F_X_ where F_1_ stands for “Formula (1)” of 0.1:1.0 *w*/*w* ratio and similarly for F_2_ and F_3_ which refer to 0.5:1.0 and 1.0:1.0 *w*/*w* ratios, respectively.

### 2.4. Synthesis of the PLGA-Cf NPs

**Figure A1:**
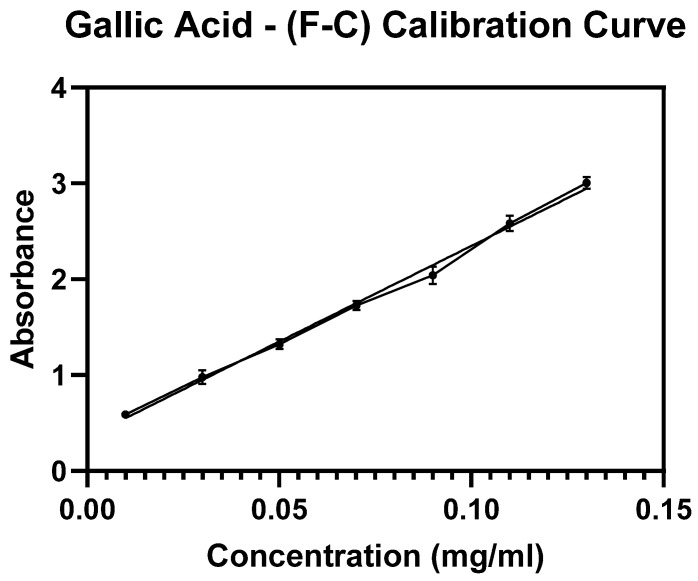
Calibration curve of GA applying F–C assay.

PLGA-Cf NPs were synthesized based on SE-SE technique according to previous literature with minute specifications [19]. First, the Cf extract was prepared according to the Solid-Liquid Extraction method (SLE) as described in literature [30] (see Appendix A [8,30,31,32,33,34,35,36,37], Table A1, Table A2 and Table A3 and Figure A1). For the preparation of PLGA-Cf NPs, PLGA was dissolved in DCM in a concentration of 50 mg/mL then mixed with an equal volume of methanol-based solution of Cf extract, achieving a ratio of 0.1:1.0 (*w*/*w*) of Cf-to-PLGA. The organic solution was added dropwise to a 20 mL aqueous solution of PVA during homogenization then transferred to ultrasonication (in ice bath). The resulted *o*/*w* nano-emulsion was subjected to magnetic stirring overnight to evaporate the organic phase. The NPs were collected by centrifugation and washed twice. The final sediment was freeze dried. The produced NPs were characterized and subjected to a second phase of optimization involving the study of impact of varying the initial drug amount in the NP on the same abovementioned responses. Accordingly, other two Cf-to-PLGA ratios were selected, 0.5:1.0 and 1.0:1.0 *w*/*w*, formulated under the identified optimum conditions and characterized.

### 2.5. Characterization of the Prepared PLGA-Cf NPs

The hydrodynamic particle size, PDI and ZP measurements were obtained using the particle size analyzer (DLS: Dynamic Light Scattering, Malvern, NANO-series, Zetasizer, Worcestershire, United Kingdom). FT-IR Spectroscopy analysis (FT-IR: Fourier Transform-Infrared Spectroscopy, using Thermo Scientific Nicolet 8700, Waltham, MA, USA) was carried out to examine the chemical composition of the unprocessed Cf extract and the formed NPs (with and without the extract) working over a range of wavenumber of 4000–400 cm^−1^. The method adopted here relied on the use of KBr-based solid pellet technique. The particle morphology and microscopic size were imaged by the FE-SEM (FE-SEM: Field Emission-Scanning Electron Microscopy, Gemini Sigma, New Philadelphia, OH, USA). Typical sample preparation required initial surface coating by sputtering with a gold film.

### 2.6. Determination of Encapsulation Efficiency (EE%) and Loading Capacity (LC%)

The amount of entrapped Cf extract within the NPs was determined indirectly based on constructing a standard calibration curve of serial concentrations of the Cf extract where the absorbance readings were detected at a maximum wavelength ($\lambda_{\max}$ = 273 nm). Therefore, the collected supernatants were subjected to Ultraviolet-Visible spectroscopy (UV-Vis, Jenway 74 series, Staffordshire, UK) (R^2^ of the calibration curve was 0.9979) for absorbance detection at the same $\lambda_{\max}$. The entrapped mass of the extract was determined using Equation (1), thereby the EE% value was calculated through Equation (2). Finally, the LC% was determined from Equation (3). This method was performed according to previously applied protocols [18,19,38,39,40,41,42].

(1) Mass Encapsulated (mg)=Initial drug mass in preparation—free drug mass

(2) $$\mathrm{Encapsulation}\;\mathrm{Efficiency}\;(\mathrm{EE}\%)=\frac{\mathrm{Mass}\;\mathrm{of}\;\mathrm{encapsulated}\;\mathrm{drug}\;\left(\mathrm{mg}\right)}{\mathrm{Initial}\;\mathrm{weight}\;\mathrm{of}\;\mathrm{drug}\;\mathrm{in}\;\mathrm{preparation}\;\left(\mathrm{mg}\right)}\;\times \;100$$

(3) $$\mathrm{Loading}\;\mathrm{Capacity}\;(\mathrm{LC}\%)=\frac{\mathrm{Mass}\;\mathrm{of}\;\mathrm{encapsulated}\;\mathrm{drug}\;\left(\mathrm{mg}\right)}{\mathrm{Total}\;\mathrm{weights}\;\mathrm{used}\;\mathrm{for}\;a\;\mathrm{preparation}\;\left(\mathrm{in}\;\mathrm{mg}\right)}\;\times \;100$$

### 2.7. Stability of TPC of Cf Extract in PLGA NPs

F-C assay was applied on the formulated NPs in order to indicate the stability of the TPC after encapsulation [8,31,32,38]. Briefly, specific equal weights of pure Cf extract, optimally prepared plain PLGA NPs and the PLGA-Cf NPs (0.1:1, 0.5:1 and 1:1) were added to 2.5 mL of 10% (*v*/*v*) aqueous F-C reagent, followed by 2 mL of 7.5% (*w*/*v*) sodium carbonate. All the prepared tubes were left in the dark for 90 min followed by centrifugation for 15 min at 20,000 rpm. Finally, supernatants were measured by UV-Vis at a wavelength of 765 nm. Gallic acid (GA) was used as a standard reference phenolic compound to which a material known for its TPC (Total Phenolic Content) is compared. Similarly, F-C assay was applied to establish a standard calibration curve using a series of known concentrations of GA. The TPC was expressed as the number of milligrams of GA that was equivalent to a gram of the extract (mg GAE/g dry extract).

### 2.8. Evaluating the Antioxidant Activity

The DPPH assay protocol was applied as elaborated previously [38] using equal predetermined weights of the NPs samples. The samples containing NPs were centrifuged and the supernatants were separated for further UV-Vis spectrometry at 517 nm. The % inhibition values were calculated using Equation (4). The NP sample that showed the highest % inhibition value among others was selected for further analyses to obtain the 50% DPPH inhibition (IC_50_) value through preparing a series of known concentrations to construct a calibration curve.

(4) $$\mathrm{DPPH}\;\mathrm{scavenging}\;\mathrm{activity}\;(\%)=\frac{A_{\mathrm{control}}-{\;A}_{\mathrm{sample}}}{A_{\mathrm{control}}}\;\times \;100$$

where $A_{\mathrm{control}}$ is the absorbance of the DPPH reagent, $A_{\mathrm{sample}}$ is the absorbance of the prepared samples.

### 2.9. Evaluating the Cytotoxic Activity

Cytotoxicity of the samples (NPs and pure Cf) were evaluated against four cancerous cell lines, MCF-7 (human breast cancer), A-549 (lung cancer), HeLa (cervical cancer) and HepG-2 (liver cancer), to assess their potential anticancer activity as well as against human normal lung fibroblast (WI-38) to assess their biocompatibility. The assessment was performed through the protocol of MTT assay as identified elsewhere [43]. All cell lines were allowed to incubate for 72 h. The numbers of viable cells and the percent cell viability were also determined, applied similarly for all types of cell lines, and calculated according to Equation (5). The relation between surviving cells and drug concentration (dose–response curve) was plotted to get the survival curve of each tumor cell line after treatment with the specified sample. The 50% inhibitory concentration (IC_50_), the concentration required to cause toxic effects in 50% of intact cells, was also reported.

(5) $$\mathrm{The}\;\mathrm{percentage}\;\mathrm{of}\;\mathrm{viability}\;(\%)=\frac{{\mathrm{OD}}_{t}}{{\mathrm{OD}}_{c}}\;\times \;100$$

where OD_t_ is the mean optical density of wells treated with the tested sample and OD_c_ is the mean optical density of untreated cells.

### 2.10. In Vitro Release and Release Kinetics Studies

The release rate of Cf from the NPs was investigated based on the quantification of the extract released from the NPs using UV-Vis spectroscopy in two different pH media (5.5 and 7.4) [39,42]. To achieve the sink condition, the release medium of pH 7.4 was prepared of 60% phosphate buffer saline (PBS) and 40% methanol (where the extract dissolves) whereas the release medium of pH 5.5 was prepared from 60% acetate buffer and 40% methanol. Typically, a dialysis bag (12,000–14,000 kDa) contained certain weight of lyophilized NPs and 5 mL of the release medium, then formed a total of 20 mL to be incubated in an orbital shaker at 37 °C and 100 rpm over a period of three days. Samples were withdrawn at predetermined time intervals throughout the 72 h and replaced by an equal volume of fresh medium. The samples were subjected to UV-Vis analysis (at 273 nm) to estimate the total cumulative amount of the extract (mg) in a unit volume of the release medium (mL). Finally, the cumulative amount of the released Cf extract was expressed as the cumulative release percent as in Equation (6). To study the kinetic profile of Cf release from the PLGA NPs, data were treated according to zero-order, first-order, Higuchi, Korsmeyer–Peppas, and Hixson–Crowell, Weibull, and Noyes–Whitney Equations [38].

(6) $$\mathrm{Cumulative}\;\mathrm{release}\%=\sum_{t=0}^{t}\frac{M_{t}}{M_{o}}\;\times \;100$$

where $M_{t}$ is the cumulative amount of the Cf extract released at each time interval while $M_{o}$ is the initial amount of the extract added to the NP formula.

### 2.11. Statistical Analysis

One-way ANOVA was carried out, followed by Tukey’s test and statistical significance was expressed at *p* < 0.05. The values of the results were reported as the mean ± standard deviation (SD) of the triplicate determinations.

## 3. Results

### 3.1. Preparation of PLGA-Cf NPs

**Figure 1:**
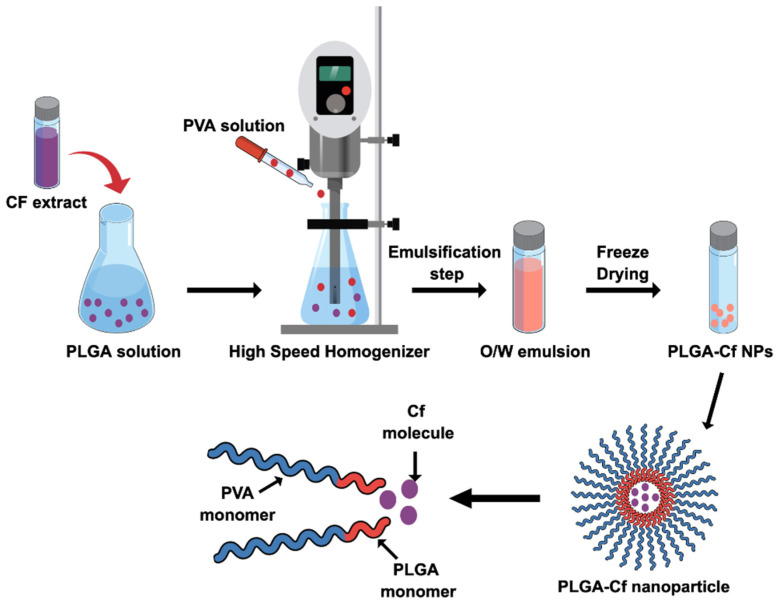
Schematic representation for PLGA-Cf NPs synthesis.

The preparation steps of PLGA-Cf NPs are illustrated in (Figure 1). As mentioned above, the PLGA-Cf NPs were prepared via an emulsification step using homogenization process followed by a freeze-drying step.

### 3.2. Statistical Analysis of the Designed Experiment

The experimental matrix from the randomized runs for the independent variables and responses is shown in (Table 2). The range of particle size (Y_1_) was 355.5 to 275.0 nm. Similarly, the ZP (Y_2_) was −20.6 to –28.5 mV. For the PDI (Y_3_), the range was 0.09–0.16. The EE% (Y_4_) was 55.1–89.3%. Finally, the LC% (Y_5_) range was from 0.55–2.54%. All responses were fitted to a quadratic model except for Y_1_ and Y_3_ which fitted to a linear model and the adequacy of these models was verified by one-way ANOVA. In the ANOVA test, the *p*-values of the models for responses Y_1_, Y_3_, Y_4_ and Y_5_ were ˂0.0001, while was 0.0009 for Y_2_. Additionally, the R^2^ values for the responses Y_1_-Y_5_ were 0.9136, 0.9812, 0.9291, 0.9961 and 0.9936, respectively. Thus, it can be concluded that all the responses fit the model well (*p* ˂ 0.05). The developed quadratic model in terms of coded values is given below:

(7) $$Y={\;\beta }_{0}+{\;\beta }_{1}X_{1}+{\;\beta }_{2}X_{2}+{\;\beta }_{3}X_{3}+{\;\beta }_{4}X_{1}X_{2}+{\;\beta }_{5}X_{2}X_{3}+{\;\beta }_{6}X_{1}X_{3}+{\;\beta }_{7}1X_{1}^{2}+\beta_{8}1X_{2}^{2}+{\;\beta }_{9}1X_{3}^{2}\;$$

where Y is the measured response, β_0_–β_9_ are regression coefficients and X_1_, X_2_, X_3_ are independent factors.

### 3.3. Analysis of Response Surfaces

**Figure 2:**
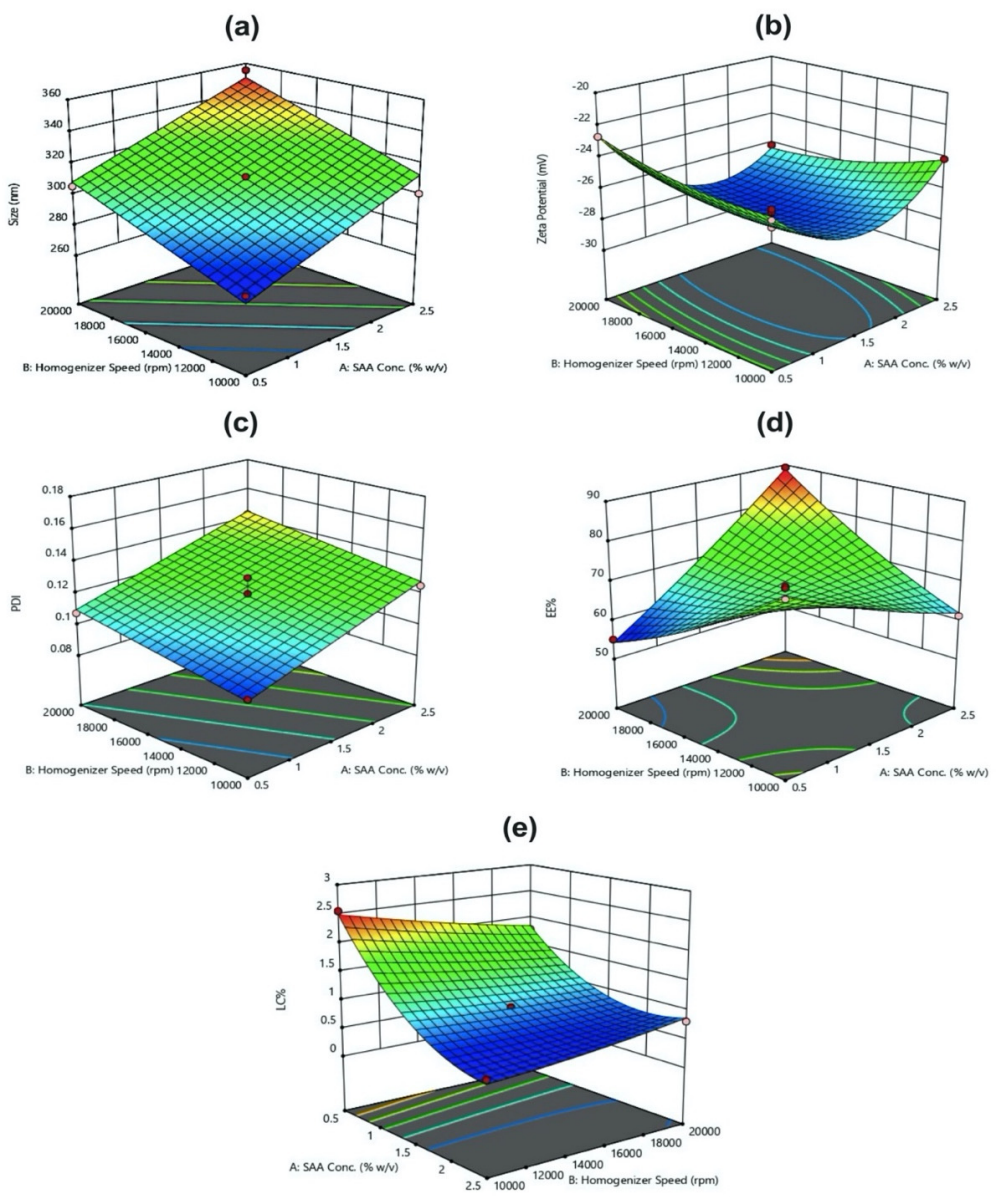
Response surface showing the effects of PVA concentration (**X_1_**) and H. speed (**X_2_**) at the mid-level of H. time (**X_3_**) on particle size (**a**), ZP (**b**), PDI (**c**), EE% (**d**), and LC% (**e**).

The effect on hydrodynamic size was represented as a linear model, where each factor affects the average size value independently from the other factors. As shown in Figure 2a, as the PVA concentration (X_1_) increased from 0.5 to 2.5% (*w*/*v*), the particle size increased. In addition, increasing the homogenization speed (X_2_) (from 10,000 to 20,000 rpm) increased the size. On the other hand, extending the homogenization time (X_3_) from 5 to 10 min resulted in an insignificant increase of the particle size.

From the 3D-surface graph in (Figure 2b), one can realize that the highest ZP value (−28.5 ± 2.690 mV) was detected when X_1_ is 1.5% (*w*/*v*), X_2_ is 15,000 rpm and X_3_ is 7.5 min. Since it is a quadratic model, this strongly suggested that all three factors might be interrelated in influencing the final ZP value. It is clearly noticed that the ZPs were negative for all the loaded NPs, which was explained by the presence of PVA at their surfaces.

Similarly, from the 3D-surface graph shown in Figure 2c, at low X_1_ (0.5% *w*/*v*), a monodisperse system was obtained. However, by increasing X_1_, high polydispersity resulted. This statistically proposed a linear relation between applying higher X_2_ and inducing polydispersity.

From Figure 2d, the maximum EE% (89.33% ± 3.925) was seen at the highest X_1_ (2.5% *w*/*v*), and the highest X_2_ (20,000 rpm) and an X_3_ of 7.5 min.

From Figure 2e, the maximum LC% (2.547 ± 0.201%) was seen at the lowest values of X_1_ (0.5% *w*/*v*) and X_2_ (10,000 rpm), but an X_3_ of 7.5 min.

#### The Optimized Formulation Conditions

To evaluate the findings of the response surface methodology, a verification run was carried out and no significant difference was found between the predicted and actual values of the five specified responses as shown in Table 3.

### 3.4. Second Phase of NPs Fabrication and Optimization

**Figure 3:**
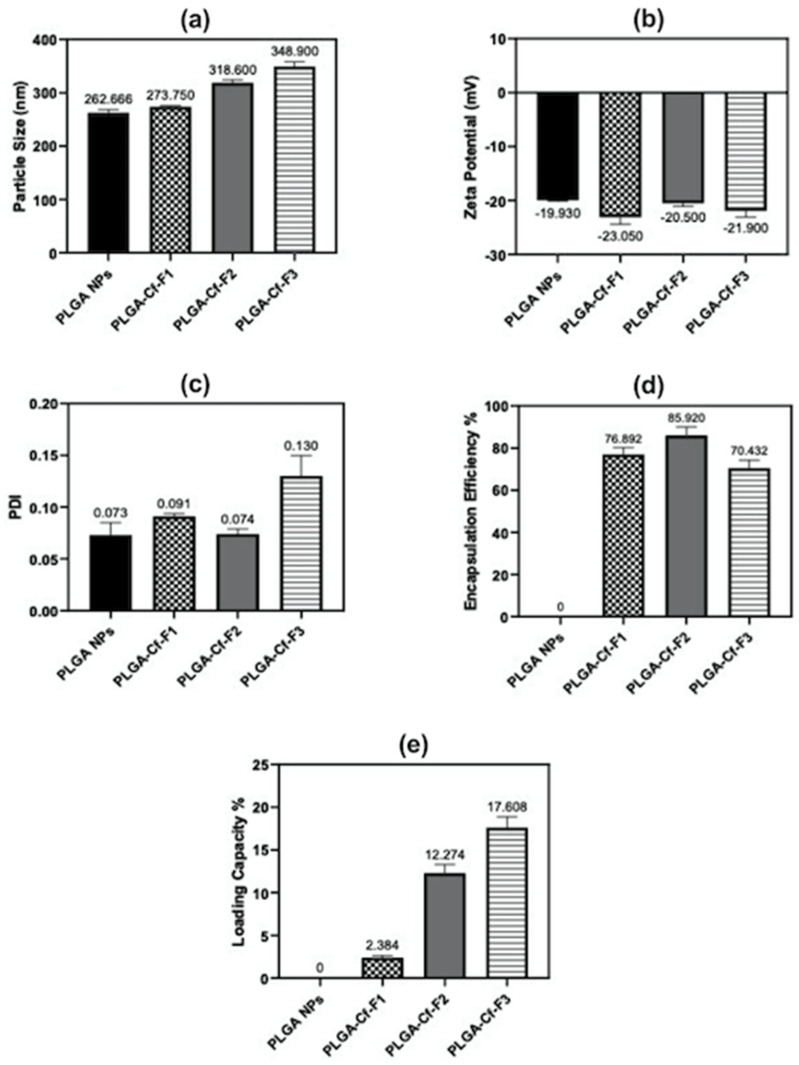
Bar charts representing the impact of increasing the initial drug amount on: average particle size (**a**), ZP (**b**), PDI (**c**), EE% (**d**) and LC% (**e**) for PLGA NPs and PLGA-Cf-F_1_, -F_2_, and -F_3_.

It could be observed in Figure 3a that the more Cf loaded into the PLGA matrix, the larger the size of the NPs obtained. Upon introduction of the drug, the size increased by 4.22, 16.38, and 33.2% for PLGA-Cf-F_1_, -F_2_, and -F_3_, respectively. For Figure 3b, the PLGA-Cf-F_1_ NPs demonstrated the highest absolute value of ZP (−23.05 ± 1.34 mV) among the loaded NPs, since they have the smallest size. The particle size distribution became less homogeneous upon increasing the drug content to the maximum limit of the experimental range as shown in (Figure 3c). It is worth mentioning that our results match previous reports [19,39]. For EE%, (Figure 3d) showed that by increasing the initial Cf amount the EE% was first increased significantly from 76.89 ± 3.17% to 85.92 ± 4.01% in F_1_ and F_2_, respectively. Surprisingly, upon further increase of the drug in F_3_, EE% showed a significant downward trend indicating that the nano-system could no longer handle higher drug ratios above the condition in F_2_. Finally, Figure 3e indicated that as the initial drug amount increased, the LC% of the nano-system increased linearly. These results were found matching with Budhian et al. [44].

### 3.5. FT-IR Spectral Analysis of the NPs

**Figure 4:**
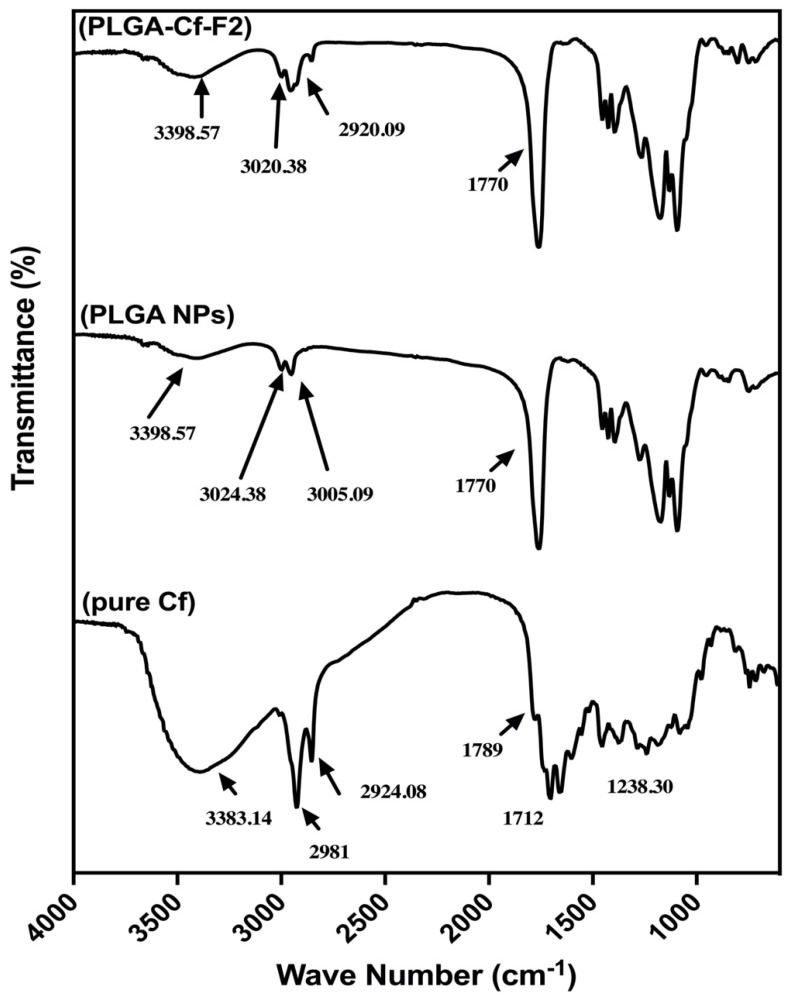
The FT-IR spectra of pure Cf, PLGA NPs, and PLGA-Cf-F_2_.

The FT-IR spectra of pure Cf, PLGA NPs and the three PLGA-Cf NPs are illustrated in Figure 4. For pure Cf, Figure 4 shows the broad and large band at 3383.14 cm^−1^ which is due to the stretching of a phenolic O–H group. The band at 2924.08 cm^−1^ strongly indicated an aliphatic stretch of C−H while the band at 1706.07 cm^−1^ represented a carbonyl moiety (C=O) which was already slightly shifted due to conjugation or a carboxylic acid. A further band found at 1600.92 cm^−1^ confirmed the conjugation since it referred to the presence of an aromatic alkene (–C=C–). Below 1600.00 m^−1^, there was a band at 1238.30 cm^−1^ which might indicate the C–O stretch while the band at 1080.14 cm^−1^ might refer to C–N stretching [41,45]. On the contrary, the FT-IR spectra of both the unloaded and loaded PLGA NPs (Figure 4, PLGA NPs and PLGA-Cf-F_2_, respectively) showed the typical bands of PLGA [46] entailing the successful and complete inclusion of Cf extract within the PLGA matrix [19,47]. The observed IR spectra of PLGA and PLGA-Cf NPs included a characteristic broad, small band of weak intensity at 3398.57 cm^−1^, indicative of a carboxylic O–H end group. The presence of several small bands at 2987.38 and 2861.09 cm^−1^ referred to the stretching vibrations of CH, CH_2_ and CH_3_. A very sharp, intense peak at 1759.08 cm^−1^ confirmed a carbonyl stretch related to the carboxylic group. From 1450 to 850 cm^−1^ there were several bands of C–H bending in relation to the spectrum having a carbonyl group (C=O). However, there was a slight deformation in the bands occurring between 3020 and 2900 cm^−1^ which was more obvious upon increasing the initial drug ratio to 0.5:1.0 and 1.0:1.0. Accordingly, the spectrum of the PLGA-Cf-F_2_ only was displayed due to the similarities with other PLGA-Cf NPs’ spectra.

### 3.6. FE-SEM Analysis

**Figure 5:**
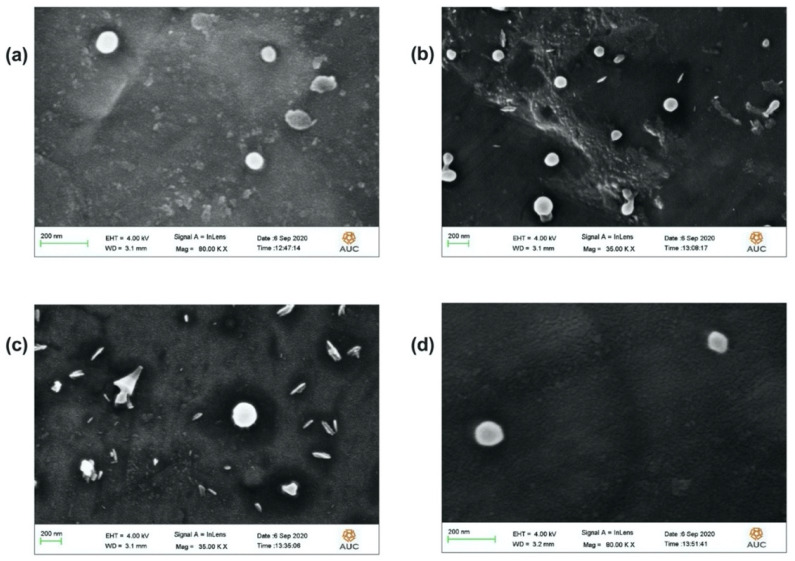
FE-SEM images of PLGA NPs (**a**), PLGA-Cf-F_1_ (**b**), PLGA-Cf-F_2_ (**c**), and PLGA-Cf-F_3_ (**d**).

The FE-SEM analysis showed circular particles (Figure 5a–d). Typically, the FE-SEM technique provided a closer look of the particles’ sizes compared to the hydrodynamic sizes provided by the DLS technique. In this context, the estimated average diameters (measured using ImageJ software) of plain PLGA NPs, PLGA-Cf-F_1_, PLGA-Cf-F_2_ and PLGA-Cf-F_3_ NPs were 73,981 ± 14,180 nm, 107,152 ± 21,582 nm, 130,005 ± 20,713 nm and 164, 487 ± 17,184 nm, respectively. These findings confirmed the increase in the average particle size with increasing the initial drug loading, which comes in agreement with the findings of the DLS technique.

### 3.7. Phenolic Compounds Content

**Figure 6:**
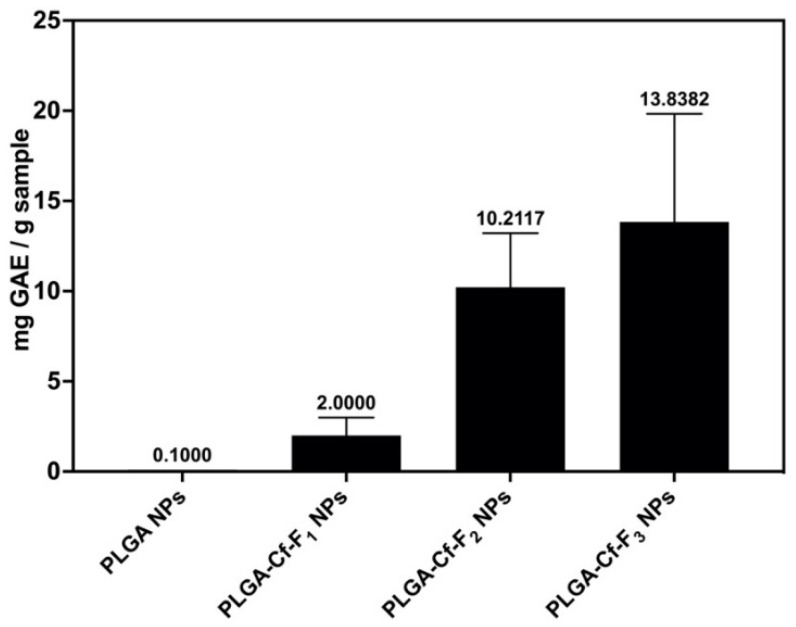
TPC of PLGA NPs and different PLGA-Cf NPs expressed in mg GAE/g sample.

The results of applying the F-C assay on the formed NPs, using 5 mg of each sample, are represented in Figure 6. The results showed an overall significance among the means. It could be observed that the unloaded NPs did not possess polyphenolic compounds, giving a zero result. Moreover, PLGA-Cf-F_3_ NPs showed the highest TPC (13.83 ± 5.99 mg GAE/g sample); however, this value was not significantly different from the TPC of PLGA-Cf-F_2_ NPs.

### 3.8. Antioxidant Activity

**Figure 7:**
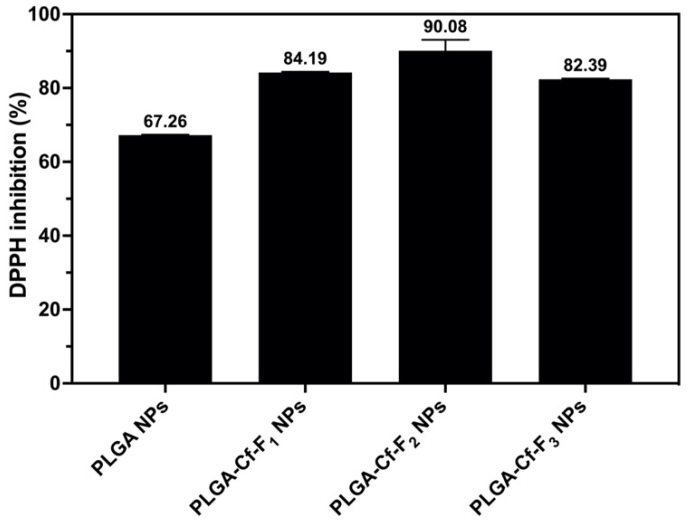
Antioxidant activity of PLGA NPs and different PLGA-Cf NPs expressed in (DPPH inhibition%).

The results of applying DPPH assay on the NPs, using 10 mg of each sample, are represented in Figure 7. From data analysis, it was found that there was no significant difference between the % inhibition value of the NP samples PLGA-Cf-F_1_ and PLGA-Cf-F_3_ (84,190 ± 0.221% and 82,397 ± 0.301%, respectively). The results indicated that the unloaded PLGA NPs possessed antioxidant activity (67,621 ± 0.239%). PLGA-Cf-F_2_ NPs showed the highest antioxidant activity (the highest % inhibition value (90,082 ± 0.199%)).

Based on all the above-matched data regarding the NP sample PLGA-Cf-F_2_, it was chosen for the remaining studies.

### 3.9. Cytotoxic Activity

**Figure 8:**
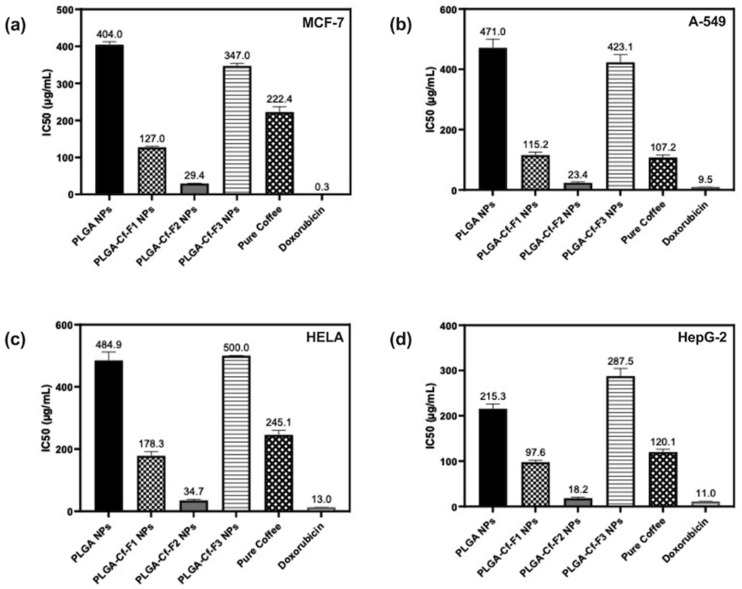
Bar charts representing the IC_50_ (µg/mL) values of the tested samples, obtained from applying MTT assay against four cancerous cell lines: (**a**) MCF-7, (**b**) A-549, (**c**) HeLa and (**d**) HepG-2.

**Figure A2:**
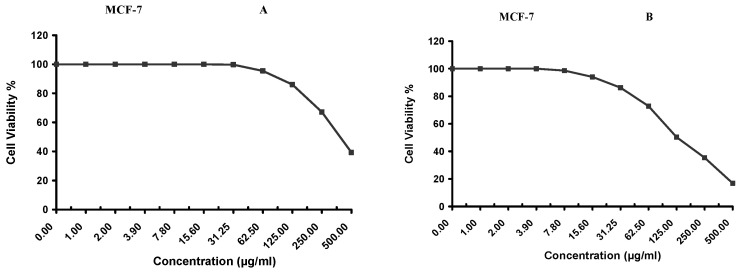

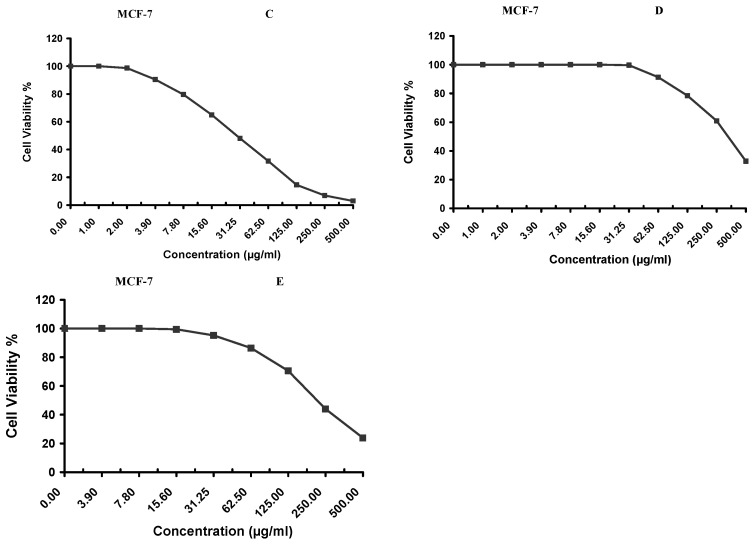
The percentage of cell viability of breast cancer cell lines (MCF-7) upon treatment with different doses of the test samples. (**A**) the unloaded PLGA NPs, (**B**) the PLGA-Cf-F_1_ NPs, (**C**) the PLGA-Cf-F_2_ NPs and (**D**) the PLGA-Cf-F_3_ NPs. Finally, graph (**E**) is for the free coffee extract.

**Figure A3:**
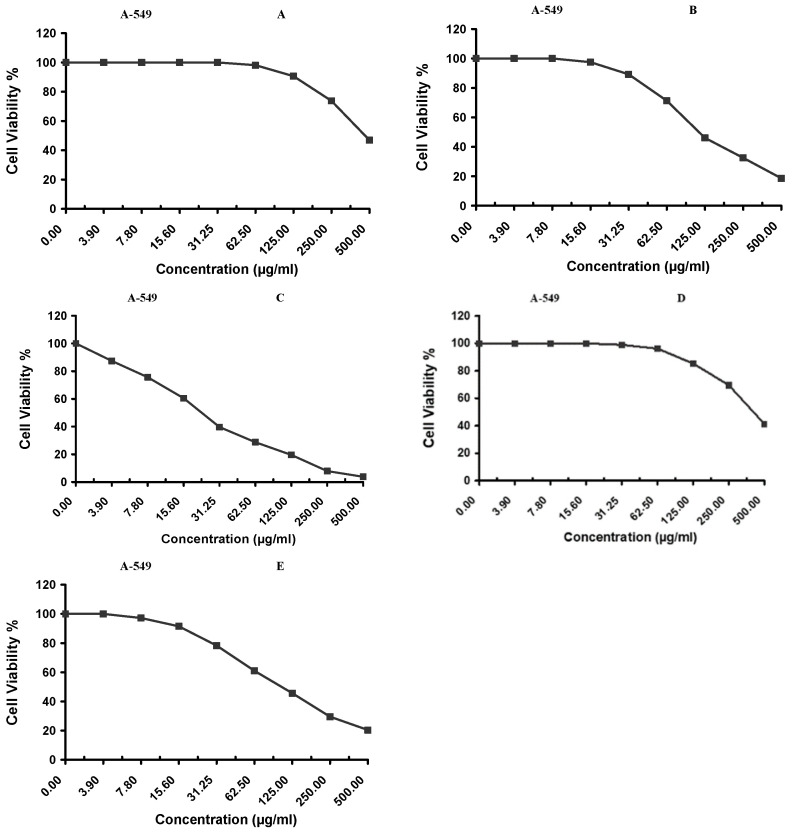
The percentage of cell viability of lung cancer cell lines (A-549) upon treatment with different doses of the test samples. (**A**) the unloaded PLGA NPs, (**B**) the PLGA-Cf-F_1_ NPs, (**C**) the PLGA-Cf-F_2_ NPs and (**D**) the PLGA-Cf-F_3_ NPs. Finally, graph (E) is for the free coffee extract.

**Figure A4:**
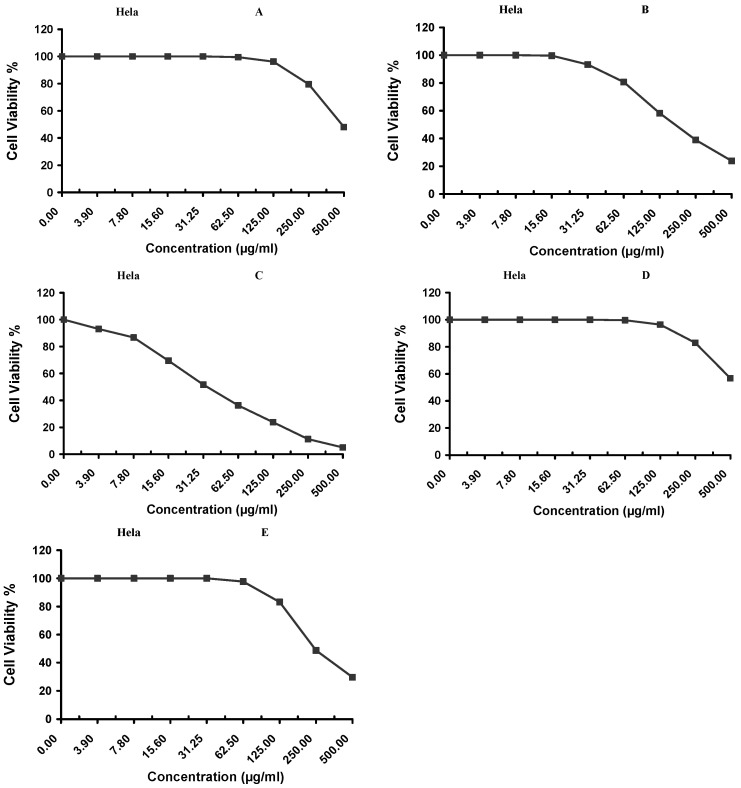
The percentage of cell viability of cervical cancer cell lines (HeLa) upon treatment with different doses of the test samples. (**A**) the unloaded PLGA NPs, (**B**) the PLGA-Cf-F_1_ NPs, (**C**) the PLGA-Cf-F_2_ NPs and (**D**) the PLGA-Cf-F_3_ NPs. Finally, graph (**E**) is for the free coffee extract.

**Figure A5:**
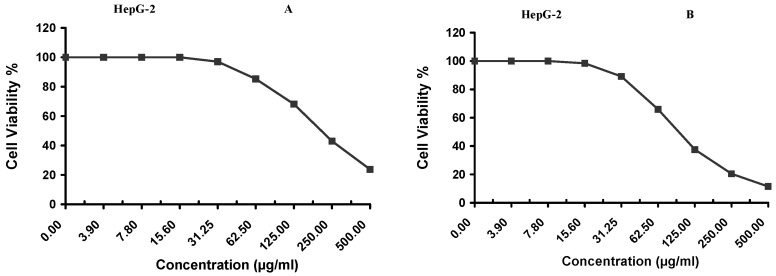

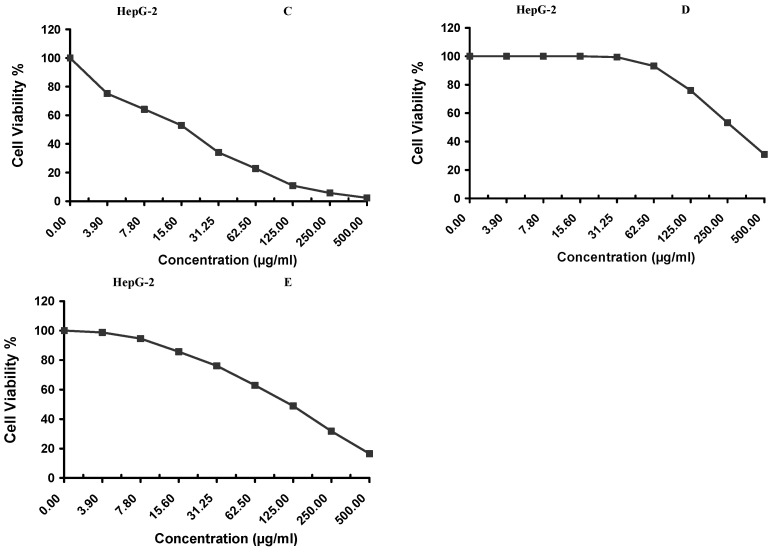
The percentage of cell viability of liver cancer cell lines (HepG-2) upon treatment with different doses of the test samples. (**A**) the unloaded PLGA NPs, (**B**) the PLGA-Cf-F_1_ NPs, (**C**) the PLGA-Cf-F_2_ NPs and (**D**) the PLGA-Cf-F_3_ NPs. Finally, graph (**E**) is for the free coffee extract.

**Figure A6:**
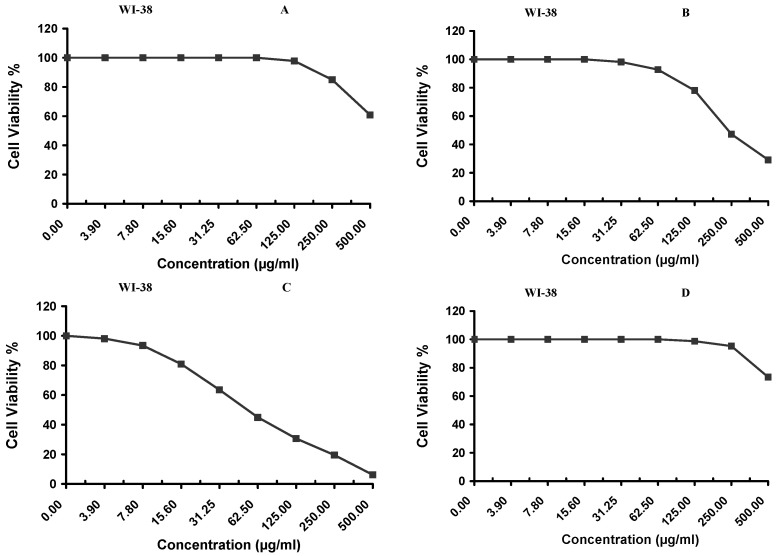

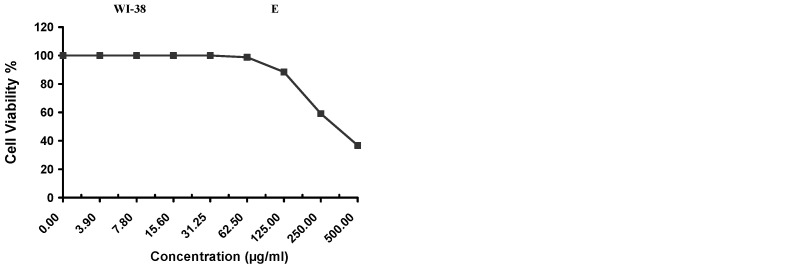
The percentage of cell viability of normal fibroblast cell lines (WI-38) upon treatment with serial concentrations of (**A**) unloaded PLGA NPs, (**B**) PLGA-Cf-F_1_, (**C**) NPs, PLGA-Cf-F_2_, (**D**) NPs, PLGA-Cf-F_3_ and (**E**) the free coffee extract.

The data on percent cell viability are provided in the Appendix B (Figure A2, Figure A3, Figure A4, Figure A5 and Figure A6); however, the IC_50_ values (µg/mL) were obtained from these data which indicated the concentration required to decrease the cells’ viability by 50%. These values of each sample against each cancer cell line type were represented in Figure 8. Doxorubicin was used as a reference (positive control) anticancer drug against MCF-7, A-549, HeLa and HepG-2 with an IC_50_ of 0.35, 9.50, 13.00, and 11.00 µg/mL, respectively. Data were statistically significant as compared to each other and to the control. Figure 8 reveals that PLGA-Cf-F_2_ NPs showed the most remarkable cytotoxic activity (the lowest IC_50_) against all types of cancerous cell lines compared to the other NP samples and the pure extract.

**Figure 9:**
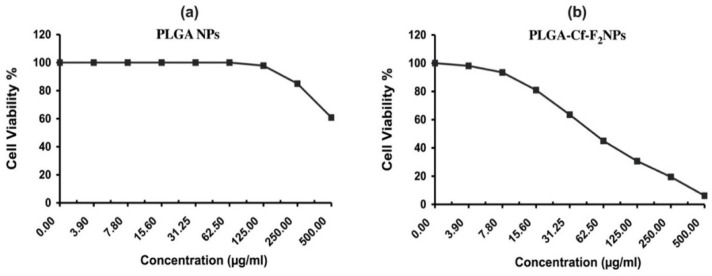
The percent cell viability of normal fibroblast cell lines (WI-38) upon treatment with serial concentrations of PLGA NPs (**a**), and PLGA-Cf-F_2_ NPs (**b**).

Furthermore, the percent cell viability of normal cell lines (WI-38) obtained for unloaded NPs and PLGA-Cf-F_2_ NPs in Figure 9 revealed two important findings: first, PLGA matrix (considered as a negative control) maintained the viability of the cells at concentrations as high as 100 µg/mL, after which the percent cell viability decreased gradually to 97.82 ± 0.64% at a concentration of 125 µg/mL. Second, the successful PLGA-Cf-F_2_ NPs showed another delayed dose-dependent cytotoxicity towards the normal cells (attributed to the effect of Cf compared to plain NPs) although that lower concentration of these NPs exhibited effective anticancer activity (IC_50_ of 29.40 ± 1.10 µg/mL against MCF-7, for instance) compared to the concentration inhibiting cell viability of normal fibroblasts (IC_50_ of 54.3.90 µg/mL).

By the end of this stage, it can be concluded that the NP sample PLGA-Cf-F_2_ combined the most desired results in terms of favorable physicochemical characteristics (small average particle diameter, uniform size distribution and the highest EE%) and remarkable biological performance.

### 3.10. In Vitro Release Study

**Figure 10:**
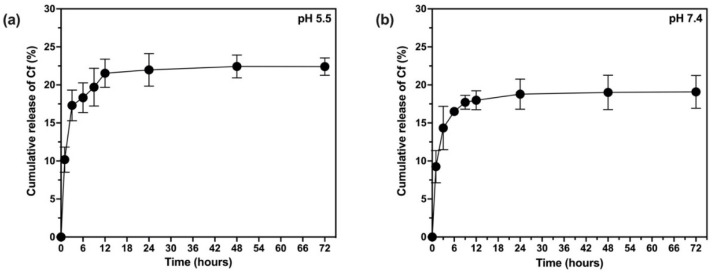
In vitro release profiles of Cf from PLGA NPs in different pH media: pH 5.5 (**a**) and pH 7.4 (**b**).

The release profile of Cf from PLGA matrix was studied for the NP sample PLGA-Cf-F_2_ in two different pH media, 7.4 (the physiological norm) and 5.5 (the cancerous cells pH) at 37 °C for 72 h. Figure 10 shows a biphasic pattern, i.e., an initial phase of fast release rate (burst) compared to a following second phase of slower release rate of the entrapped extract (noticed in several previous studies [48]). At pH 5.5 ≈ 20.263 ± 1.947% was released after 6 h followed by a sustained release in the next hour, reaching to 19.012 ± 1.145% after 72 h. The profile for the release medium at pH 7.4 was similar to that of pH 5.5; however, ≈18.664 ± 0.158% of the drug released after 6 h followed by a slower rate in the next 72 h, reaching to ≈16.925 ± 2.158%.

**Figure 11:**
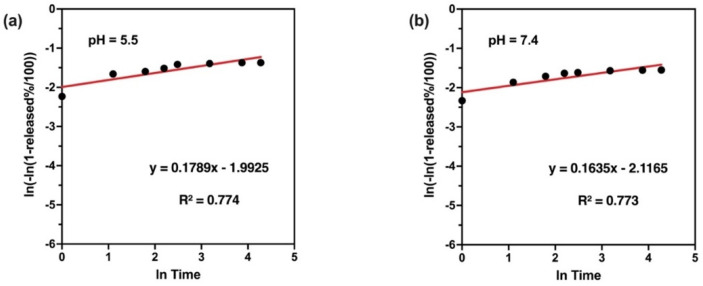
Weibull kinetic plots of Cf released from PLGA NPs in different pH media: pH 5.5 (**a**) and pH 7.4 (**b**).

To understand the mechanism of drug release from PLGA matrix, the release data were fitted into zero, first, Higuchi, Hixson–Crowell, Noyes and Whitney, Weibull, and Korsmeyer–Peppas models as shown in Table 4. It was found that in vitro Cf release from PLGA matrix was best fitted in the Weibull release model as indicated by highest value of coefficient (R^2^) as shown in Figure 11.

## 4. Discussion

### 4.1. Preparation of PLGA-Cf NPs

In the SE-SE method, emulsification and stabilization of the nano-droplets are crucial factors. Primarily, the external energy source provides shear stresses to the organic phase resulting in the formation of nano-droplets, where the emulsifier (PVA) plays an important role in the emulsification process and in protecting the particles from agglomeration and coalescence. The PVA chain is formed from alternating hydrophilic and hydrophobic segments which provide the adsorption “arms” at the NPs’ interface, i.e., upon dissolution of PVA, it partially hydrolyses and reorients, in the presence of organic entities, such that the hydrophobic moieties interconnect with the PLGA chains to create a matrix, while the hydrophilic moieties face the aqueous phase. Thereby, PVA can exert its stabilizing effect via reducing the aggregation of NPs and thereby lowering their size [49].

### 4.2. Analysis of Response Surfaces

The multiple effects of the three independent variables were studied on the five selected responses as follows.

#### 4.2.1. Effects on Hydrodynamic Size

As mentioned above and shown in Figure 2a, a linear mode of impact was seen upon increasing PVA concentration from 0.5 to 2.5% (*w*/*v*) where the particle size increases. This might be due to the viscosity enhancement of the aqueous phase that reduces the net shear stress available for droplet breakdown and subsequently increased the particle size [50]. In addition, the particle size increase, along with increasing the homogenization speed from 10,000 to 20,000 rpm, could be explained by the high number of collisions leading to excessive particles’ breakdown and hence generating very high surface area which could not be stabilized by the available surfactant [26].

#### 4.2.2. Effects on Zeta Potential

Regarding the impact of PVA concentration on the ZP values, at low X_1_ (0.5% *w*/*v*), ZP was decreased to −21.50 ± 0.550 mV (as illustrated in Figure 2b), particularly at slightly higher homogenization speed and longer duration, since exerting higher net amount of shear stresses would excessively breakdown the NPs, thereby exposing greater surface area that was inefficiently covered by the low surfactant amount available [51].

#### 4.2.3. Effects on Polydispersity Index

Inhomogeneity of particle size distribution became more prominent upon increasing PVA concentration (X_1_) from 0.5 to 2.5% (*w*/*v*) (as shown in Figure 2c) which might result from the removal of important amounts of PVA during the repeated washing processes which could cause some agglomeration of the NPs [48]. Furthermore, a linear trend was detected upon increasing the homogenization speed, thus polydispersity was detected; that could be attributed to the creation of larger-sized NPs from aggregates of smaller-sized ones together with the presence of other medium-sized NPs [48].

#### 4.2.4. Effects on EE%

From Figure 2d, the EE% increased linearly when applying high net shear forces (X_2_ and/or X_3_) during emulsification which might be due to the fact that a unidirectional and less turbulent flow in the case of lower net shear forces might result in the loss of the extract from the organic phase [52] (this effect dominated the effect of X_1_).

#### 4.2.5. Effects on LC%

The LC% of NPs was governed, to a great extent, by the partitioning of the drug between the polymeric organic (dispersed) phase and the aqueous (continuous) phase and its subsequent separation from the continuous phase and deposition on the NP surface [51].

### 4.3. Second Phase of NP Fabrication and Optimization

Figure 3a strongly indicated the presence of the compounds to be encapsulated would majorly lead to increasing the organic phase viscosity and this would make it more difficult to disperse the two phases during the emulsification process and eventually originate larger particles [39]. In Figure 3b, the presence of Cf extract in PLGA NPs always potentiated the ZP probably due to the presence of negatively charged molecules that might be adsorbed on the NP surface [53].

### 4.4. FT-IR Spectral Analysis of the NPs

As depicted in Figure 4, the disappearance of the characteristic absorption peaks of Cf in the IR spectra of the loaded PLGA NPs strongly confirmed the well encapsulation and shielding of the extract within the polymeric matrix, as previously observed by Kızılbey et al. [19] and Chereddy et al. [47].

### 4.5. Phenolic Compounds Content

The insignificant increase of the TPC in PLGA-Cf-F_3_ over that in PLGA-Cf-F_2_ might be attributed to the physical adsorption of phenolic compounds by the surface-free alcoholic hydroxyl groups of PVA, through hydrogen bonding and hydrophobic interactions, rather than its encapsulation compared to the EE% and LC% of PLGA-Cf-F_2_ NPs. Thus, these surface-bound phenolic compounds were easily desorbed, indicating a higher value, although it was not significant [54].

### 4.6. Antioxidant Activity

As the results show in Figure 7, there was greater inhibition of DPPH by the extract-loaded PLGA compared to the free extract, and this impact might be attributed to the protection of the active compounds by the PLGA from any free radicals that could be produced during the incubation period of the assay. The improvement in the antioxidant activity upon nano-encapsulation within PLGA was reported in the literature (Pereira et al. [39] and Pool et al. [55]).

### 4.7. Cytotoxic Activity

By analyzing the data, we confirmed the anticancer potentials of Cf in safe doses (due to the polyphenolic acids, phenolic compounds and alkaloids) [41,56]. The increase in the cytotoxicity of Cf by nano-encapsulation within PLGA matrix provided three additional mechanisms over the non-encapsulated form, i.e., the selective cellular uptake (endocyto-sis) of the PLGA NPs into the cancerous cell lines (reported previously with different types of cancer cell lines such as breast (MCF-7) [42] and lung cancers (A-549) [57]), the im-proved release of the extract from the NPs (since MTT assay was performed on pure Cf and PLGA-Cf suspended in water), and finally, the inhibition of efflux transport (*p*-glycoprotein) by PLGA, allowing more extract to be safely internalized and indwelled [57]. In addition, the NP sample PLGA-Cf-F_2_ was extraordinarily the most efficient anticancer among other nano-particulate counterparts owing to multiple merits, i.e., having the highest EE% (85,920%), the size distribution uniformity (PDI of 0.074) that is advantageous in providing equal drug diffusion rates from the equal-sized NPs, in addition to the lower ZP value (−20.500 mV) which came in agreement with previous studies suggesting that such low range of surface charges could augment the uptake of the NPs into the cancer cells besides its effective cellular inhibitory effect [58]. Collectively, these results are strongly encouraging for its possible therapeutic use as an anticancer agent or as a synergistic agent to reduce the dose of other chemotherapeutic agents, hence minimizing their toxic side effects.

It is no wonder that this finding came in agreement with previous reports studying other plant herbal materials, such as that by Betbeder et al. [59]. However, we cannot construct a fair comparison between the different types of studied plant-based nano-formulations regarding their cytotoxic activities due to the wide range of variables controlling their potentials: starting from the native anticancer potential of the plant material/constituent, the type of synthesis method applied, the numerous process and formulation factors, the resulting physicochemical parameters of the NPs, the release study conditions and release kinetics, the conditions of the MTT assay, etc. For instance, we can numerically demonstrate the superiority of our extract-based nano-system over an ethanolic extract of Polygala senega (EEPS)-encapsulated PLGA NPs against A-549 cell line [60], which reported a 77.46% inhibition of cell viability at 200 µg/mL, compared to a higher viable cell inhibition of 80.43% at a 37.5% lower concentration (125 µg/mL) by our NPs, PLGA-Cf-F_2_. By looking at the conditions of NP fabrication and in vitro testing, we could find that the implemented method was the solvent displacement technique, adopting F68 (polyoxyethylene-poly-oxypropylene) as the stabilizer, which resulted in spherical NPs of sizes less than 150 nm, ZP of −31.6 ± 2.5 mV, PDI of 0.273 ± 0.012 and EE% of 80%, while the incubation period of the MTT assay was 24 h. These differences can strongly explain the lower cytotoxic potential of the EEPS-encapsulated PLGA NPs compared to our nano-system. Another study by Paul et al. [61] reported the cytotoxic activity of Chelidonium majus alkaloid (chelidonine)-encapsulated PLGA NPs against the HepG-2 cell line with an IC_50_ value of 10.22 µg/mL (within 48 h incubation) compared to 18.20 µg/mL (within 72 incubation) of our PLGA-Cf-F_2_ NPs. The physicochemical characteristics of the chelidonine-encapsulated PLGA NPs included spherical NPs of a size range of 123 ± 1.15 nm, ZP of −19.6 ± 2.48 mV and EE% of 82.6 ± 0.574%, which were produced by applying the solvent displacement technique and stabilized by F68. In addition to these points of difference, the higher cytotoxic potential detected for this nano-system could be attributed to the faster release behavior which showed a 60% drug release within the first 48 h (at pH 7.4) compared to ≈19% of coffee release within a longer time of 72 h, at pH 7.4 (as discussed in the next section). On the other hand, ergosterol-loaded PLGA NPs were fabricated, by Zhang and his group [58], using emulsion-solvent evaporation method (with some modifications) and PVA as the stabilizer which produced spherical NPs of diameter range 156.9 ± 4.8 nm, PDI of 0.08 ± 0.018 and ZP of −19.27 ± 1.13 mV. These NPs were incubated for 72 h in the MTT assay and reported IC_50_ values of 9.43 µg/mL and 4.70 µg/mL against MCF-7 and HepG-2 cell lines, respectively, compared to 29.4 µg/mL and 18.2 µg/mL of PLGA-Cf-F_2_ NPs against MCF-7 and HepG-2, respectively. The greater cytotoxic activity of PLGA-ergosterol NPs could possibly be explained by the release rate of the drug which reported ≈32% release within 72 h at pH 5, compared to ≈23% coffee release within the same time period and pH range. As mentioned above, an important factor to be considered while studying the enhancement degree of the cytotoxic potential of a plant material is its native cytotoxicity, as seen with the PLGA-chelidonine NPs against MCF-7, where nano-encapsulation induced a 46.63% increase (approximately half the enhancement (86.78%) that was seen in our nano-system) over the free chelidonine. In addition, with the PLGA-ergosterol NPs against MCF-7, the particle size reduction within polymeric matrix induced only a 19.95% activity improvement relative to the free ergosterol, in addition to a 26.79% increase in the activity against HepG-2, compared to an 84.84% increase of the cytotoxicity of coffee when in polymeric nano-system relative to its free form.

### 4.8. In Vitro Release Study

Basically, the drug release was a result of a combination of factors, i.e., penetration of the release medium into the particle matrix, diffusion of extract through the matrix, and the erosion, swelling and degradation of the polymer. Being that the degradation of PLGA polymer was slow, the initial faster release of the extract would therefore largely depend on the fast diffusion of the extract found close to or attached to the surface of the NPs which passed between the polymer chains into the external medium. The affinity of Cf ex-tract for the release medium (40% methanol:60% release medium) could be another reason for its fast release. The uniform release profile (with decreased release rate) noticed following the fast effect could be explained by the slower diffusion of the extract found in the innermost of the PLGA matrix, which travelled through a progressively longer diffusion path, requiring a longer time to release [19,48,62,63,64,65].

As seen in Figure 11 and Table 4, the in vitro release mechanism was best fitted in the Weibull release model as indicated by the highest value of coefficient (R^2^). In the case of medium molecular weight PLGA, NP release is mainly mediated through the diffusion process with very little contribution from degradation. PLGA with medium molecular weight showed far more sustained release. Due to extremely high affinity of the drug with the PLGA, the drug was very slowly diffusing out of the polymer matrices and showed a much-sustained release [66].

## 5. Conclusions

The present study evidently demonstrated a successful encapsulation of coffee extract into the biopolymeric nano-system, PLGA/PVA via the SE-SE method (owing to the hydrophobic nature of the extract). Primarily, the influence of three independent factors (PVA concentration, homogenization speed and duration) on the particle diameter, PDI, ZP, EE% and LC% was evaluated and statistically analyzed by BBD to identify the optimum formulation conditions. The results of this phase were very close to the expected values (with an average particle size of 273.750 nm, a PDI of 0.091, a ZP of −23.050 mV, a 76.892% as EE% and a 3.383% as LC%), confirming the success of the established design in fabricating the nano-system. By characterizing the NPs, the optimum formulation conditions were achieved at a PVA concentration of 0.5% (*w*/*v*), at a homogenization speed of 10,000 rpm for a duration of 7.5 min. A secondary phase was conducted to study the influence of increasing the initial drug loading on the aforementioned responses. Characterizing the NPs suggested a full and tight shielding of the drug by PLGA, and spherically shaped NPs with slight irregularity upon increasing the initial drug amount with higher TPC. In vitro evaluation of the NPs showed that sample PLGA-Cf-F_2_ (of particle size 318.60 ± 5.65 nm, PDI 0.074 ± 0.015, ZP −20.50 ± 0.52 mV, EE% 85.92 ± 4.01%, LC% 12.274% and a loading ratio of 0.5:1) recorded the most desirable results among other NPs and the pure Cf, in terms of the highest antioxidant activity (90,082 ± 0.199% inhibition), and the highest cytotoxicity against the four selected cancer cell lines (with an $IC_{50}$ of 29.40 ± 1.10 µg/mL against MCF-7 cells, 23.40 ± 3.20 µg/mL against A-549 cells, 34.70 ± 4.10 µg/mL against HeLa cells and 18.20 ± 2.60 µg/mL against HepG-2 cells) with preferential toxicity over the normal fibroblasts (WI-38). The in vitro release rate study was performed on the succeeded NP sample at pH 5.5 and pH 7.4 for three days and showed a biphasic release rate pattern in both pH media. Finally, we can conclude that nano-encapsulation is a very promising tool for stabilizing the physicochemical properties of plant-sourced extracts as coffee extract, and for enhancing their respective biological activities. Accordingly, it can pave the way for endless applications in the pharmaceutical, food and cosmetic sectors.

## Figures and Tables

**Table 1:** Variables and constrains in Box–Behnken experimental design.

	Level			
**Independent variables**	−1	0	1	Constrains
**X_1_: PVA conc. (*w*/*v*%)**	0.5	1.5	2.5	In the range
**X_2_: homogenization speed (rpm)**	10,000	15,000	20,000	In the range
**X_3_: homogenization time (min)**	5	7.5	10	In the range
**Dependent variables**				
**Y_1_: Particle size (nm)**				Minimize
**Y_2_: ZP (mV)**				Maximize
**Y_3_: PDI**				Minimize
**Y_4_: EE (%)**				Maximize
**Y_5_: LC (%)**				Maximize

**Table 2:** Experimental matrix and observed responses from randomized runs in BBD.

	Independent Variable			Dependent Variable				
Run	PVA (% *w*/*v*)	H. Speed (rpm)	H. Time (min)	*p*. Size (nm)	ZP (mV)	PDI	EE (%)	LC (%)
**1**	1.5	10,000	10	297.466	−23.90	0.096	70.740	0.996
**2**	0.5	20,000	7.5	305.400	−22.70	0.107	55.120	1.778
**3**	2.5	15,000	5	327.433	−23.25	0.161	70.377	0.634
**4**	1.5	15,000	7.5	305.200	−28.50	0.120	68.320	0.962
**5**	1.5	20,000	5	332.466	−24.80	0.141	61.572	0.867
**6**	2.5	15,000	10	339.333	−23.00	0.112	83.560	0.752
**7**	2.5	10,000	7.5	300.766	−24.10	0.125	61.280	0.552
**8**	1.5	15,000	7.5	311.700	−27.50	0.118	68.907	0.890
**9**	1.5	15,000	7.5	299.300	−27.30	0.130	67.440	0.949
**10**	0.5	15,000	5	281.700	−20.60	0.111	66.810	2.155
**11**	0.5	15,000	10	291.900	−21.50	0.088	69.200	2.232
**12**	0.5	10,000	7.5	275.000	−24.40	0.093	78.980	2.547
**13**	2.5	20,000	7.5	355.500	−26.15	0.143	89.330	0.804
**14**	1.5	20,000	10	332.433	−24.65	0.113	84.746	1.193
**15**	1.5	10,000	5	301.766	−24.55	0.120	76.240	1.073

**Table 3:** The optimized PLGA-Cf NPs observed and predicted response values.

Independent Variable	Optimized Level	
**X_1_: PVA Conc. (%*w*/*v*)**	0.5	
**X_2_: H. speed (rpm)**	10,000	
**X_3_: H. time (min)**	7.493	
**Overall desirability**	0.796	
**Dependent variables**	**Expected**	**Observed**
**Y_1_: Particle size (nm)**	270.506	273.750
**Y_2_: Zeta Potential (mV)**	−24.193	−23.050
**Y_3_: PDI**	0.093	0.091
**Y_4_: Encapsulation Efficiency (%)**	79.425	76.892
**Y_5_: Loading capacity (%)**	2.488	2.383

**Table 4:** Kinetics data of Cf release from PLGA NPs in different pH mediums (pH 5.5 and pH 7.4).

Release Kinetics Model	pH 5.5		pH 7.4	
	K	R^2^	K	R^2^
**Zero order**	0.102	0.392	0.080	0.367
**First order**	0.017	0.156	0.016	0.153
**Hixson–Crowell**	0.002	0.401	0.001	0.374
**Weibull**	1.196	0.774	1.178	0.773
**Korsmeyer–Peppas**	1.789	0.534	1.738	0.534
**Noyes–Whitney**	1.001	0.406	1.001	0.378
**Higuchi**	1.212	0.579	0.964	0.557

Where R^2^ is the regression coefficient, and K is the model release constant.

## Data Availability

The data presented in this study are available in the Appendix A and Appendix B.

## Appendix A. Extraction of the Phenolic Compounds from Coffee

### Appendix A.1. Extraction Method

Basically, the initial solid-to-liquid ratio of coffee to the extracting solvent was selected based on a ratio of 1:5 (*w*/*v*), respectively, as already done previously [30]. During the extraction process, two factors, the solvent type (four solvents i.e., distilled water, methanol/water 50:50 (*v*/*v*), methanol/water 80:20 (*v*/*v*) and absolute methanol) and temperature (at room temperature (RT) and at 55 °C ± 5 °C, which was specifically chosen to be below the boiling point of methanol (64.7 °C) and also to avoid breaking down of the phenolic compounds), were varied in order to investigate their effects on the resulting extracts in terms of the TPC and the antioxidant power.

Typically, a certain amount of the roasted coffee grounds was weighed and allowed to soak in the proper volume of each of the four different solvent systems to achieve the pre-specified ratio. For each solvent type, two samples were prepared, one of which was left at RT (20 °C) while the other was put at higher temperature (55 °C ± 5 °C) and controlled by a thermometer in a hot water bath. All of the eight samples were labeled (as mentioned in Table A1) and maintained on magnetic stirring (at 500 rpm) for 90 min. After that, all the eight samples were subjected to a primary filtration using a qualitative filter paper (medium speed, grade 102, 9° cm, maximum pore size 15–20 µm) to obtain a primary filtrate of each. A final filtration was done using a nylon syringe filter (pore size 0.45 µm). Finally, the filtrates were subjected to freeze drying.

**Table A1:** Eight coffee samples were identified as follows: (sample labeling was abbreviated by using the initials of the solvent system in addition to the number of the higher ratio in the system (if any), dash, then the temperature condition applied (RT or h (for heating)).

No.	Conditions of Extraction		Sample Label
	Type of Solvent	Temperature	
1.	Absolute Methanol	RT	M-RT
2.	Absolute Methanol	55 °C ± 5 °C (heat (h))	M-h
3.	Methanol:Water 50:50	RT	M50W-RT
4.	Methanol:Water 50:50	55 °C ± 5 °C	M50W-h
5.	Methanol:Water 80:20	RT	M80W-RT
6.	Methanol:Water 80:20	55 °C ± 5 °C	M80W-h
7.	Distilled water	RT	W-RT
8.	Distilled water	55 °C ± 5 °C	W-h

### Appendix A.2. Assay for TPC

F–C assay was carried out on all the prepared coffee extracts as a primary method to indicate the TPC in each sample, as described previously [8,31,32,38]. Started by preparing a 1 mg/mL solution of each of the eight extracts followed by applying the assay in triplicates where 0.5mL of each extract solution was added. To all, 2.5 mL of 10% (*v*/*v*) aqueous F–C solution was added, left in the dark and shaken for 5 min, then 2 mL of 7.5% (*w*/*v*) ${\mathrm{Na}}_{2}{\mathrm{CO}}_{3}$ was added to each. All the prepared tubes were left in the dark on the shaker (at 350 rpm) for 90 min. After that, the tubes were placed in the centrifuge for 15 min. at 20,000 rpm. Finally, the obtained supernatant was spectrophotometrically measured at a wavelength of 765 nm. Gallic acid (GA) was used as a standard reference phenolic compound to which a material known for its TPC is compared. Similarly, F–C assay was applied to establish a standard calibration using a series of known concentrations of GA (from 0.01 to 0.13 mg/mL). The TPC was expressed as the number of milligrams of GA that was equivalent to a gram of the extract (mg GAE/g dry extract).

Gallic acid (GA) was used as a standard reference phenolic compound. Similarly, F–C assay was applied to establish a standard calibration using a series of known concentrations of GA (from 0.01 to 0.13 mg/mL). A straight-line equation was obtained from the GA calibration curve which aided in calculating the concentration (in mg/mL) of the TPCs in each extract sample using (Equation (A1)). Subsequently, the TPC was expressed as the number of milligrams of GA that was equivalent to a gram of the extract (mg GAE/g dry extract) by multiplying the calculated concentrations by 1000 in order to convert it into masses.

y = ax + b (A1)

where a is the slope, b is the intercept and y refers to the absorbance readings for the extract samples, so, x obtains the corresponding concentration for each absorbance.

### Appendix A.3. Assay for Antioxidant Activity

DPPH assay was applied to the prepared coffee extract samples [8,31,32,38]. The assay protocol was applied according to Brand-Williams, Cuvelier Berset [33] with some modifications. Typically, 0.1mL of each extract solution (0.1 mg/mL) was used. To each of the eight tubes, 3 mL of 0.1 mM DPPH methanol-based reagent was added, left in the dark and shaken for 45 min at 350 rpm. Finally, spectrophotometric measurements were done at a wavelength of 517 nm (two blank tubes were prepared i.e., one included absolute methanol while the other blank included distilled water while the control tube contains the DPPH reagent). Based on the absorbance readings, the antioxidant potential of a sample was obtained in the form of percent inhibition (% inhibition, according to Equation (A2)) where the sample with the highest value of % inhibition indicates the most powerful antioxidant activity among other samples.

(A2) $$\mathrm{Inhibition}\;\mathrm{percent}\;\mathrm{of}\;\mathrm{DPPH}\;(\%)=\frac{A_{\mathrm{control}}-{\;A}_{\mathrm{sample}}}{A_{\mathrm{control}}}\;\times \;100$$

$A_{\mathrm{control}}$ is the absorbance of the DPPH reagent, $A_{\mathrm{sample}}$ is the absorbance of the prepared coffee extracts. Moreover, GA was used as a standard reference antioxidant for comparative investigation of the antioxidant power of the tested extract samples. So, similarly, the DPPH assay was applied to GA to establish a calibration curve using predetermined serial concentrations (from 0.01 to 0.13 mg/mL). Another characteristic value can be obtained from the curve (${\mathrm{IC}}_{50}$) which was the GA concentration that was capable of neutralizing half (50%) the initial concentration of DPPH and was used for future comparative analysis against the best coffee extract and the optimized NPs.

### Appendix A.4. Results of Applying F–C Assay

Quantification of the collected absorbance data (at $\lambda_{\max}\;=$ 765 nm) of the coffee extracts was performed by a linear regression from a calibration curve of GA constructed using the range of concentrations specified earlier. Then the straight-line equation obtained from the curve aided in calculating the universal unit of expression, mg GAE/g sample, which refers to that a single gram of the tested sample is equivalent to a certain (calculated) weight (in mg) of the reference polyphenolic substance. Accordingly, (Figure A1) shows the straight line obtained by a nonlinear regression of the GA absorbance readings for each concentration where the R^2^ (coefficient of determination = 0.9956) indicates that 99.56% of the variability of absorbance readings are around its mean.

To obtain the GA equivalence per 1 g of the extract sample, the calculations were done using the constants produced by the straight-line equation (Equation (A3)) i.e., the slope (=19.964) and the intercept (=0.3517) which were obtained as follows:

y = 19.964x + 0.3517 (A3)

Based on the fact that (y axis) is substituted by the value of the average absorbance for every coffee sample, the (x axis) is the calculated corresponding concentration of the sample (mg/mL) in equivalence to GA (mg/mL).

Similarly, the rest of the values of the remaining coffee extract samples were obtained. In this sense, Table A2 shows the final results of performing the F–C assay on the prepared coffee samples.

**Table A2:** The results of F–C assay expressed as average mg GAE/g sample.

Sample	Average mg GAE/g Sample	±SD
M-RT	97.07	3.44
M-h	121.76	2.50
M50W-RT	75.02	1.77
M50W-h	84.30	2.20
M80W-RT	90.62	2.14
M80W-h	112.98	3.12
W-RT	55.73	0.91
W-h	57.44	0.58

As a conclusion of this section: by analyzing the reported data through applying one-way ANOVA, the outcomes have shown very high significance among the means (*p* ˂ 0.0001 and R^2^ value of 0.9931). Upon further data analysis based on multiple comparisons, the results limit the decision to one sample only, M-h (121.76 ± 2.50 mg GAE/g sample), since it recorded the highest mean value indicating that it contained the highest TPC among other coffee extract samples.

### Appendix A.5. Results of Applying DPPH Assay

The results obtained from applying the DPPH assay to the prepared eight coffee extract samples were tabulated in (Table A3) that demonstrated the resulted mean values of the % inhibition values.

**Table A3:** The results of DPPH assay expressed as percentage inhibition.

Sample	% Inhibition	±SD
M-RT	70.68	0.39
M-h	77.72	0.32
M50W-RT	58.61	0.38
M50W-h	61.87	0.53
M80W-RT	68.54	1.42
M80W-h	74.72	1.58
W-RT	48.84	1.51
W-h	48.85	0.94

By applying an ordinary one-way ANOVA for analyzing the collected data, a statistical significant difference can be revealed among the means with a probability value (*p* ˂ 0.0001) indicating a very high confidence (with greater than 99.9999%) in the applied protocol and the resulted data. Also, the R^2^ value is 0.9936.

Additionally, through multiply comparing the results between every value against the rest of the values, significant differences were also observed in most of the cases. Moreover, this significance validates the effects of the solvent type and the extraction temperature on the resulting antioxidant activities of the extracts as observed with their respective TPC (which was evidenced in previous work such as with Ghafoor et al. [34], Vergara-Salinas et al. [35] and Akowuah et al. [36]). It is important to mention that the difference between the mean values of the samples M-h and M80W-h showed a significance (77.72 ± 0.32 and 74.72 ± 1.58% inhibition, respectively) although it is very low (P value is 0.0389).

As reviewed in previous literature, the antioxidant activity usually correlates to the polyphenolic compounds and phenolic acids in a sample material which are extracted as a direct consequence of the used solvent and applied temperature [8,31,32,37].

As a conclusion, with reference to the findings of F–C and DPPH assays which pointed out sample M-h with the highly remarkable outcomes among other samples. Accordingly, it was the sample of choice to be used in the NPs preparation. But first, an analysis of its chemical composition was provided by the FT-IR spectroscopy technique in order to be used as a reference when compared with its nano-encapsulated counterparts.

## Appendix B. In Vitro Evaluation of the Prepared PLGA-Coffee NPs

### Cytotoxic Activity

The data on % cell viability is presented for each type of the tested cancer cell lines in Figure A2, Figure A3, Figure A4 and Figure A5 as well as for the normal fibroblast cell line in Figure A6.
